# PARP1 rs1805407 Increases Sensitivity to PARP1 Inhibitors in Cancer Cells Suggesting an Improved Therapeutic Strategy

**DOI:** 10.1038/s41598-019-39542-2

**Published:** 2019-03-01

**Authors:** Irina Abecassis, Andrew J. Sedgewick, Marjorie Romkes, Shama Buch, Tomoko Nukui, Maria G. Kapetanaki, Andreas Vogt, John M. Kirkwood, Panayiotis V. Benos, Hussein Tawbi

**Affiliations:** 1 Division of Hematology/Oncology, University of Pittsburgh School of Medicine, Pittsburgh, Pennsylvania USA; 2 Department of Computational and Systems Biology, University of Pittsburgh School of Medicine, Pittsburgh, Pennsylvania USA; 3 Joint Carnegie Mellon University-University of Pittsburgh PhD Program in Computational Biology, Pittsburgh, Pennsylvania USA; 4 Division of Pulmonary, Allergy and Critical Care Medicine, Department of Medicine, University of Pittsburgh, Pittsburgh, Pennsylvania USA; 5 Drug Discovery Institute, University of Pittsburgh School of Medicine, Pittsburgh, Pennsylvania USA; 6 Department of Melanoma Medical Oncology, Division of Cancer Medicine, University of Texas MD Anderson Cancer Center, Houston, Texas USA; 7 Department of Investigational Cancer Therapeutics, Division of Cancer Medicine, University of Texas MD Anderson Cancer Center, Houston, Texas USA

## Abstract

Personalized cancer therapy relies on identifying patient subsets that benefit from a therapeutic intervention and suggest alternative regimens for those who don’t. A new data integrative approach, based on graphical models, was applied on our multi-modal –omics, and clinical data cohort of metastatic melanoma patients. We found that response to chemotherapy is directly linked to ten gene expression, four methylation variables and PARP1 SNP rs1805407. PARP1 is a DNA repair gene critical for chemotherapy response and for which FDA-approved inhibitors are clinically available (olaparib). We demonstrated that two PARP inhibitors (ABT-888 and olaparib) make SNP carrier cancer cells of various histologic subtypes more sensitive to alkylating agents, but they have no effect in wild-type cells. Furthermore, PARP1 inhibitors act synergistically with chemotherapy in SNP carrier cells (especially in ovarian cancer for which olaparib is FDA-approved), but they are additive at best in wild-type cancer cells. Taken together, our results suggest that the combination of chemotherapy and PARP1 inhibition may benefit the carriers of rs1805407 in the future and may be used in personalized therapy strategies to select patients that are more likely to respond to PARP inhibitors.

## Introduction

Advances in cancer management have improved the overall outlook of patients with metastatic malignancies but chemotherapy remains a mainstay of treatment for most common cancers. Virtually all patients develop resistance to chemotherapy after prolonged exposure given the first order kinetics of cytotoxics that generally cannot eradicate cancer. Understanding the mechanisms of this resistance presents new opportunities to improve the therapeutic index of cytotoxic agents and identify novel drug targets.

A large proportion of cytotoxic agents exert their effect through DNA damage. Thus, DNA repair pathways constitute cells’ main resistance mechanisms and potential drug targets. Base excision repair, a predominant pathway for single strand break (SSB) damage repair, utilizes a family of related enzymes termed poly-(ADP-ribose) polymerases (PARP), which are activated by DNA damage^1^. Given the critical role of PARP1 in base excision repair, PARP inhibition emerged as a therapeutic target and early studies demonstrated dramatic potentiation of chemotherapeutic agents in the presence of PARP inhibition^2,3^. Recent evidence indicates that, in addition to the catalytic inhibition of PARP activity, PARP inhibitors (PARPi) induce cytotoxic PARP-DNA complexes through PARP “trapping” that augment the cytotoxicity of alkylating agents. It is therefore of utmost importance to identify molecular features that act not only as biomarkers for patient stratification but also offer insights into the mechanisms of resistance to chemotherapy. Metastatic melanoma remains an excellent model for chemotherapy resistance given its refractory nature, despite the fact that current management of metastatic melanoma is mostly based on non-chemotherapy based strategies (e.g., targeted and immune-based therapies).

In this study, we used a probabilistic graphical method we have developed, *CausalMGM*^4–6^, in high-throughput data from a cohort of metastatic melanoma patients on chemotherapy. We identified a number of features directly linked to chemotherapy response, including a SNP in the PARP1 gene that is highly predictive of resistance to chemotherapy. Subsequently, *in vitro* studies investigated the impact of this PARP1 variant on PARPi sensitivity and demonstrated its utility as a predictive biomarker. Given the role of PARP1 in DNA repair, we propose this SNP as a characteristic biomarker for PARPi sensitivity to guide patient selection for chemotherapy treatment alone or in combination with PARPi.

## Materials and Methods

### Melanoma study design

Using a retrospective cohort study design (Table 1), we evaluated 66 patients with metastatic melanoma who were treated with alkylator-based chemotherapy at the Melanoma Center of the University of Pittsburgh Cancer Institute (UPCI) between 2000 and 2007. Patients were identified through the institution’s medical record data repository. All methods for data collection and subsequent experiments were carried out in accordance with relevant guidelines and regulations. All experimental protocols were approved by the University of Pittsburgh Institutional Review Board (IRB number: PRO10090257). To meet HIPAA guidelines and ensure patient confidentiality, all data were de-identified (De-ID Software, University of Pittsburgh) using an honest broker system. Frozen tissues were available from metastatic lesions on 18 patients and formalin-fixed paraffin embedded tissues from 51 patients. Only pre-treatment tumor specimens were included in this analysis. In addition, chemotherapy regimens studied were primarily single-agent dacarbazine (DTIC), single-agent temozolomide (TMZ) or DTIC-based combinations (including CVD, Cisplatin + Vinblastine + DTIC). Response to chemotherapy was defined as documented objective tumor regression upon treatment. Patients with disease progression after 2 cycles of chemotherapy or with stable disease lasting less than 4 months were considered non-responders.

**Table 1:** Characteristics of study population.

	N	Median (Range)
Age in years	66	51 (23–90)
Survival (months)		16 (2–88)
	**N**	**Percent**
Gender		
Female	21	32%
Male	45	68%
Response		
Responder	18	27%
Non responder	48	73%
Types of chemotherapy		
Single agent TMZ	29	44%
TMZ-based combination	3	5%
Single agent DTIC	16	24%
DTIC-based combination	18	27%

### Gene Expression and methylation data collection

Total RNA was isolated from melanoma tissues using the PerfectPure RNA Tissue kit (5Prime Inc., MD, USA). RNA was quantified using Ribogreen RNA quantitation Kit (Molecular Probes, Eugene OR) and its quality was evaluated by RNA Integrity Number using the Agilent Bioanalyzer. Whole genome gene expression analysis was carried out using the Illumina HT-12 Expression BeadChip (Illumina, San Diego, CA) which targets > 25,000 genes (>48,000 probes) derived from the RefSeq (Build 36.2, Rel 22) and UniGene databases^7^.

DNA was isolated from melanoma tissues using ArchivePure DNA Cell/Tissue kit (5Prime Inc., MD, USA). DNA samples (0.5 μg) were treated with sodium bisulphite using the EZ DNA methylation Gold kit (Zymo Research, Irvine, CA), and bisulphite-treated DNA was applied to an Illumina Infinium HumanMethylation27 BeadChip (Illumina, San Diego, CA) for DNA methylation profiling. This microarray permits the quantitative measurement of DNA methylation for 27,578 CpG dinucleotides spanning 14,495 genes. Methylation status of the interrogated CpG sites was determined by comparing β-values, the ratio of the fluorescent signal from the methylated allele to the sum from the fluorescent signals of both methylated and unmethylated alleles.

### Single Nucleotide Polymorphism (SNP) Panel Selection

In order to be cost-effective we decided to create a custom-made melanoma/cancer Illumina SNP panel. First, we performed literature search and used expert knowledge of the current cancer and melanoma biology to select relevant pathways and their corresponding genes. In this step, the following pathways were considered: DNA repair, MAPK, AKT, PI3K and mTOR signaling, immune response, DNA methylation, cell cycle, apoptosis, and signal transduction via erB signaling. We found 65 genes in these pathways and we retrieved all SNPs from dbSNP in the ±10 Kbp region around each of them. SNP filtering followed, which included the following criteria: (1) minor allele frequency (MAF) <10%; (2) screening using the Illumina Assay Design Tool (Illumina, San Diego, CA) to ensure SNPs can be incorporated in an optimal custom array design. The remaining SNPs of each gene were in strong LD (in general, r^2^ ≥ 0.8). We selected ~6 SNPs per gene (where possible) to complete the 384 SNP Illumina design (Supplementary Table S1). After data collection, quality control and initial analysis further reduced this dataset to 118 SNPs for the following reasons: no variation observed across samples, poor SNP call rate (less than 90%) or failed Hardy-Weinberg Equilibrium.

### TaqMan Single Nucleotide Polymorphism (SNP) Genotyping Assay

Genomic DNA was extracted from the melanoma tumor or the full panel of cancer cell lines using QIAamp DNA Mini kit (Qiagen, Calencia, CA) and subjected to SNP Genotyping using a Taqman SNP Genotyping assay (C__11639200_20, SNP ID: rs1805407) (Applied Biosystems^TM^, Waltham, MA). Each reaction contained 12.5 µL TaqMan Universal PCR Master Mix, 0.625 µL TaqMan SNP Genotyping Assay, 10.875 µL distilled water and 1 µL gDNA (10 ng/µL), with a final reaction volume of 25 µL. Positive control samples for the three possible genotypes were included with each genotyping reaction. In addition, at least two no template (negative) controls were included.

### Cell lines

A panel of human tumor cell lines originating in five common cancers was used: melanoma (FEMX, A375, M14, MW-852, WM-115, SK-Mel-31, SK-Mel-2), colon (HT-29, LoVo, SW620, HCT-116, LS174T), breast (MCF-7, MDA-MB-231), ovarian (SKOV-3, A2780) and lung (A549, Calu-6, H-460, H522). HT-29, HCT-116 and H-522 cell lines were generously provided by Dr. John Schmitz (all at HCC); Dr. Jian Yu (LoVo and SW620); Dr. Vera Levina (SKOV-3, H460, and A549); Nupur Gangopadhyay (Calu-6); Dr. Lisa Butterfield (MDA-MB-231, MCF-7, LS174T and M14); Dr. John Kirkwood (FEMX); Dr. Anda Vlad (Magee Woman Cancer Institute) (A2780); Dr. Hassane Zarour (SK-Mel-2); WM-115, A375, WM-852 and SK-MEL-31 were purchased from American Type Culture Collection (ATCC). All cell lines were cultured according to their recommendations (in RPMI-1640 or DMEM medium supplemented with 10% FBS) without antibiotics and routinely tested for Mycoplasma. Cell lines were grown in monolayer at 37 °C in a humidified atmosphere containing 5% CO_2_ and routinely sub-cultured twice weekly. Cell lines were fingerprinted at the University of Pittsburgh Cell Culture and Cytogenetics facility to establish identity.

### MTT Assays

Cytotoxicity was assessed using the MTT colorimetric dye reduction assay, according to the manufacturer’s instructions (TACS® MTT Cell Proliferation Assay (MTT-CPA) from Trevigen, Inc.). Briefly, cells were seeded in triplicates in 96 wells at appropriate cell density (optimized for each individual cell line to have exponentially growing cells during the course of the assay), in their respective regular culture medium. After 24h, medium was aspirated and cells were incubated with increasing concentrations of test agents for 72h at 37 °C. The MTT Reagent was subsequently added to each well and the plates were incubated for another 4h at 37 °C in the dark. In each well, 100 µl of Detergent Reagent was then added and incubated at 37 °C until complete solubilization of formazan crystals. Cell viability was measured by reading the absorbance at 570 nm using a Safire 2 microplate reader (Tecan, Männedorf, Switzerland). All values were normalized to vehicle treated control wells. The values obtained for each of the triplicates were averaged, and IC_50_ values were defined as the concentrations of drug(s) that inhibited growth by 50% relative to controls.

### Cell proliferation assays

RPMI 1640 and Dulbecco’s modified Eagle medium (DMEM) with L-Glutamine, cell culture media were purchased from Cellgro (Mediatech, Inc.). Heat-inactivated Foundation Fetal bovine serum (FBS) was purchased from Gemini bio-products. The PARPi veliparib (ABT-888, Abbott Laboratories, Abbott Park, Illinois, USA) used in the cell proliferation assays was generously provided by Dr. Jan Beumer (Hillman Cancer Center, HCC). The ABT-888 used in the drug interaction assays was purchased from Selleck Chemicals LLC (Houston, TX, USA). ABT-888 and olaparib were dissolved in dimethyl sulfoxide (DMSO) to a stock solution of 100 mM. Working solutions were then prepared in PBS for subsequent serial dilutions. The final concentration of DMSO in drug-treated cultures was always less than 0.5% (v/v) and did not contribute to cytotoxicity (data not shown). MMS, an alkylating agent that results in DNA damage at the same bases as dacarbazine and temozolomide and that is used extensively as an alternative in the laboratory setting, was purchased from Sigma.

### MMS toxicity in the presence of fixed amounts of PARPi

The cytotoxic effects of the alkylating agent MMS, alone or in combination with PARP inhibitors veliparib (ABT-888) or olaparib were assessed by MTT assay (see above). Cells were treated with increasing concentrations of MMS either alone (0 to 1 mM) or in the presence of a fixed concentration of PARPi (veliparib: 10 nM, olaparib: 5 nM) for 72h at 37 °C. Data were normalized to vehicle alone (0.5% DMSO) for MMS treatments, or 10 nM ABT-888 or 5 nM olaparib, for combination treatments. Half-maximal inhibitory concentration (IC_50_) values of MMS alone or in combination with PARPi were calculated by curve fitting in Graph Pad Prism (GraphPad Software, Inc., Ver 5.0, San Diego, CA). IC_50_ values from three independent experiments were averaged and expressed as mean ± standard deviation (S.D.). A *chemopotentiation factor* was defined as the ratio between IC_50_s of MMS in the presence or absence of PARPi. Cells were classified as “resistant” if their potentiation factor (ratio) was less than 1, and “sensitive” if the ratio was >2. For each cell line, *p*-values were calculated using the Student t-test (two-tailed, paired) to compare the IC_50_ means of each treatment group (MMS alone or MMS + PARPi).

### Quantitative analysis of MMS/PARPi interactions by median effect analysis

Interactions between PARPi (ABT-888 or olaparib) and MMS were investigated by the Chou and Talalay median effect principle^8^. To accommodate this analysis, on each day of experiments, cells were treated with ABT-888 or MMS (to establish IC_50_ values) and fixed ratio combinations thereof based on equipotency of the two test agents. Five two-fold dilutions ranging from one fourth of the IC_50_ to four times the IC_50_ of each drug in combination plus a control were tested in three independent experiments with duplicate samples. MTT data were then analyzed using the Compusyn Software (version 1.0, ComboSyn Inc.; Paramus, NJ, USA), which provides a convenient tool to perform median effect analysis as described by Chou and Talalay^8^. The program calculates a combination index (CI) for each actual data point from the combination curves that is used to identify synergistic, additive, and antagonistic drug interactions. The program also calculates predicted CI values for every effect level (affected fraction or Fa), which when plotted as a function of Fa serve to visualize and quantify the effects of drug combinations over the entire dose range. CI values of 1, <1, and >1 represent additivity, synergism, or antagonism, respectively. Predicted CI values of three independent experiments were averaged and analyzed by one-sample Student’s *t*-test for significant deviation from additivity (i.e., CI = 1).

### Integrative analysis of omics data and outcome variables

With the term *Mixed Graphical Models* (MGM) we refer to graphical models that are learned over variables of mixed type, i.e., continuous and discrete variables. We used CausalMGM, an algorithm we recently developed^4–6^ and used for biomarker discovery^9^, to learn a directed graph over the variables in our dataset, which consisted of continuous (gene and miRNA expression, DNA methylation) and discrete (single nucleotide variants (SNPs), response to TMZ treatment) variables. The resulting directed graph selected those variables that are directly connected to response to TMZ treatment. We used subsampling to identify stable edges as we describe previously^4^. This is standard procedure, since the number of independence tests required is exponential to the number of variables.

## Results

### Identifying predictive markers of treatment for metastatic melanoma patients

**Figure 1:**
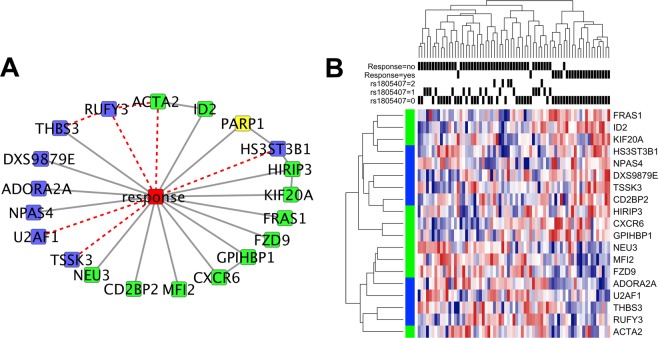
Conditional Gaussian sub-network around response to treatment with edge filter (**A**). Blue nodes represent methylation probes, green nodes represent mRNA expression probes and yellow nodes represent SNPs. Dashed red lines indicated edges removed by filtering step. Heatmap of variables directly connected to response to TMZ treatment (**B**). Black bars show rs1805407 status and response to treatment. Rows marked with green on the left are mRNA expression profiles and those marked with blue are methylation profiles.

Our dataset consisted of gene and microRNA expression, DNA methylation, SNP data from a selected panel and the clinical variable “response to TMZ”. We used CausalMGM to learn a network over the top 1000 features most correlated (pairwise) with “*response”* clinical variable, a binary variable indicating response/no response to TMZ treatment dichotomized at presence of a response or stability of disease at 4 months of therapy. The 1,000 most correlated features in the input dataset included 557 mRNA expression probes, 425 methylation probes, 14 miRNA probes and 4 SNPs. BRAF mutation status was also included in the input variables to see if it had an effect on any of the features linked to *response*, although its direct correlation with it in this dataset was poor (R^2^ = 0.025). The largest interconnected output network included 20 features directly connected to the *response* variable in our initial (undirected) learned network (Fig. 1A). We emphasize that the identified features are connected to the *response* variable not only because they have high pairwise correlation with it (Fig. 1B), but also because they contain unique information of response even when conditioned on all other variables or subsets of variables in the dataset. Because of this property, such direct associations are frequently referred to as “causal”^10,11^, although in biological applications this term should be used with caution. From the 20 features initially connected to response, 15 were left connected after filtering by edge orientation (Fig. 1A, black lines). A methylation feature for DXS9879E (LAGE3) is one of them, and it has been linked to survival in non-small cell lung cancer^12^. Notably, this important feature is not present in the top 20 (pairwise) correlated features with *response*, as other (indirect) association yield higher pairwise correlation values. ID2 expression is also directly linked to *response*. ID2 is known to induce growth and proliferation in squamous cell carcinoma^13^. No miRNA was found to be directly linked to TMZ response in our dataset.

### SNP rs1805407 in PARP1 is strongly associated with worse outcome in melanoma patients

PARP1 SNP rs1805407 is the only SNP in our dataset that is directly linked to *response*. We found this SNP to be an excellent predictor of worse outcome since all 21 patients that had the SNP (C/T or C/C) showed no response to TMZ treatment (Supplementary Figure S1) (*p*-value = 4.6e-5). Because of the strong direct association, the role that PARP1 plays in repairing TMZ mediated DNA damage, and the availability of PARP inhibitors, we decided to further investigate this finding. rs1805407 is located on the 2^nd^ intron of PARP1, ~4 Kbp downstream of the PARP1 transcription start site and 35 bp downstream of the 3’ splice site of exon 2. Since this SNP does not appear to have any functional or regulatory role, we used SNAP^14^ to find other SNPs in strong linkage disequilibrium (LD) with it. The CEU population panel of 1000 Genomes pilot 1 contained 51 variants in perfect LD (R^2^ = 1) (Supplementary Table S1) with rs1805407. Of these, two are upstream of the transcription start site (TSS). Specifically, rs6665208 is 3,573 bases upstream of the PARP1 TSS and overlaps with ENCODE ChIP peaks for MAFF and MAFK, which are both related to blood cancers^15^. rs2077197 is 238 bases upstream and overlaps peaks for AP-2α, CTCF, HA-E2F1, ZBTB7A, Pol2, CEBPB and YY1. Many of these factors are related to cancer. For example, AP-2α and ZBTB7A are known tumor suppressors^16,17^. CEBPB plays a role in senescence of prostate cancer cells^18^ and in multi-drug resistance^19^. E2F1 is induced by DNA damage^20^. CTCF is an insulator protein and YY1 participates in long-range chromosomal interactions. According to GTEx eQTL database, *all these SNPs are significantly associated with lower PARP1 expression* in whole blood (*p*-value = is 10^−6^ to 10^−7^, depending on the cell type and the SNP; see also Supplementary Figure S2).

Another SNP (rs1805405) is also in perfect LD with rs1805407 (R^2^ = 1) and is annotated as a ‘Splice region variant’ because it is located 5 bp upstream from the splice site between intron 2 and exon 3. Finally, we found a strong dependence between rs1805407 and two other SNPs that have been previously associated with melanoma susceptibility: rs3219090 (D′ = 1, R^2^ = 0.43^21^ and rs2249844 (D′ = 1, R^2^ = 0.46)^22^. Due to high LD values with rs1805407 none of the above SNPs was included in our dataset.

### SNP rs1805407 is related to decreased cytotoxicity of alkylating agents in cell lines with the variant

Given that rs1805407 is associated with worse outcome of melanoma patients treated with TMZ and that PARP1 has a critical role in repair of DNA lesions caused by TMZ, one plausible hypothesis is that rs1805407 is either associated with increased PARP1 expression and/or activity or decreased PARP1 trapping after treatment with alkylating agents^23–25^. We looked for more insights into the role of rs1805407 in cell response to various drugs. The NCI-60 Cell Miner database^26^ contains the response of 60 cell lines to ~50,000 compounds. We evaluated whether drugs affect differentially the cell lines that have at least one copy of rs1805407 (C/T or C/C) *vs* WT (T/T). The Affymetrix 500k SNP arrays used by Cell Miner did not include rs1805407, so we used the *k*-nearest neighbors method with three of the 51 perfectly correlated variants with probes in the array (rs1073991, rs10799349 and rs3219027) to infer the rs1805407 genotype in each cell line. Analysis of the IC_50_ values in cell lines predicted to have at least one allele of rs1805407 (n = 23; C/T or C/C) *vs* wild type (n = 37; T/T) showed statistically significant resistance or sensitivity to four compounds, three of which are alkylating agents with action similar to TMZ (TMZ is not included in the NCI-60 dataset) (Supplementary Table S2). These results are compatible with the hypothesis that SNP rs1805407 (or one of the 51 SNPs in perfect LD with it) may cause increased PARP1 expression and/or activity thus helping to repair the damage caused by TMZ; or decreased PARP1 trapping potentially eliminating the additional cytotoxic effects of PARP-DNA complexes induced by PARP inhibition.

### SNP rs1805407 is related to PARP inhibitor potentiation of alkylating agent cytotoxicity

Experimental validation of the association of a SNP to increased PARP1 activity in patient-derived tissues is not straightforward because PARP1 is an inducible enzyme and its activity may depend on the timing of the biopsy with respect to prior therapies. Alternatively, one can use cell lines to test whether PARP1 inhibition affects the response to alkylating agents in an SNP-dependent way. This can be done by blocking PARP1 after treatment with an alkylating agent in cells with or without the SNP.

Literature search identified 15 cell lines (Supplementary Table S3) from various tumor types, which had reported activity of alkylating agents alone or in combination with a PARPi (CEP-6800, AG14361, NU1085, NU1025 or ABT-888)^27–32^. We used qRT-PCR to confirm the rs1805407 genotypes in these cell lines. We classified cell lines as “resistant” to the combination of TMZ with PARPi if the reported experiments did not enhance cytotoxicity of TMZ when a PARPi was added; likewise, cell lines that showed significant enhancement of TMZ cytotoxicity with PARPi were classified as “sensitive”. Six of nine cell lines that had at least one C in position rs18050407 were sensitive, and all six cell lines that were WT (T/T) were resistant (*p* = 0.003, G test).

**Figure 2:**
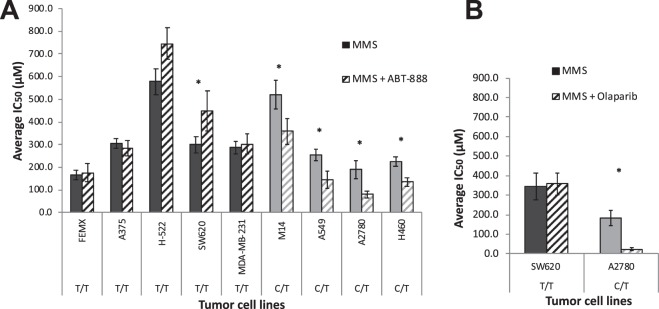
PARP1/SNP genotype is predictive of MMS + PARPi combination treatment efficacy. Plot of IC_50_ values. **(A)** ABT-888 and (**B**) olaparib treatment. *MMS*: alkylating agent used; *ABT-888/olaparib*: PARP1 inhibitors used; *left bars*: MMS only; *right bars*: MMS + PARPi. *Dark grey bars:* wild type (T/T) for rs1805407; *light grey bars:* heterozygotes (C/T). *Star* indicates that combination treatment (MMS + PARPi) has significantly different effect than alkylating agent alone (*p* < 0.05, Student’s t-test, paired two-tailed).

Although these are intriguing observations, the information used in the analysis was collected from different laboratories that used different PARPi to determine sensitivity or resistance. Therefore, we performed similar experiments on nine cell lines from various histologies (melanoma, lung, colon, ovarian, and breast cancer): five WT (T/T) and four C/T for SNP rs1805407 (Supplementary Table S4**)**. MMS was used as alkylating agent and ABT-888 was used to inhibit PARP activity. All four SNP cell lines were found to be significantly more sensitive to the combination treatment in agreement with our hypothesis; while in all five WT cell lines the combination treatment had no potentiation effect (Fig. 2A). Notably, for the WT cell line SW620 ABT-888 significantly increased the IC_50_ of MMS suggesting potential antagonism.

We extended those results to olaparib treatment of two cell lines: A2780 (SNP) and SW620 (WT); and we observed similarly significant potentiation of MMS cytotoxicity with the addition of olaparib in A2780 (Fig. 2B). Specifically, the average IC_50_ value for MMS alone was 182.43 (±24.02) µM and for MMS + olaparib was 39.22 (±8.80) µM (*p*-value = 0.02). By comparison, the corresponding values for SW620 (WT) cells were not significant: 342.71 (±68.67) μM with MMS only and 357 (±55.57) μM with MMS + olaparib (5 nM). (*p*-value = 0.72) (Supplementary Table S4).

### PARP inhibitor and alkylating agents exhibit synergy in relation to SNP rs1805407 and antagonism in relation to the wild-type genotype

We investigated potential synergistic and antagonistic effects of alkylating agent (MMS) and PARPi (ABT-888) on cell lines with different rs1805407 genotypes. Exponentially dividing cells were exposed for 72h to increasing concentrations of ABT-888 (0–500 µM) or MMS (0–1 mM) (single drug treatment) or combined at a fixed ratio based on their corresponding IC_50_ value (drug combination treatment). Cell survival was assessed by MTT assay. We then determined the Chou-Talalay combination index (CI)^8^ of ABT-888 with MMS in four established cell lines: two with the variant C/T (A2780-ovarian cancer, M14-melanoma) and two WT T/T (SW620-colon, H522-lung cancer). Although CI is not a statistical measure, it nevertheless provides insight into whether the effect of both agents is additive (CI = 1), synergistic (CI < 1), or antagonistic (CI > 1). Both SNP carrier cell lines (A2780, M14) exhibited strong and moderate effect, respectively across different concentrations (Table 2, Supplementary Figure S3). In WT cell lines the effect is additive at best (H522). Interestingly, the SW620 cell line had decreased MMS cytotoxicity after the addition of ABT-888. This might indicate antagonism as the C.I. was consistently higher than 1 across the tested concentrations. All graphs of the cytotoxic effects of MMS alone or in combination with PARPi are presented in Supplementary Figure S3. Bliss analyses showed the same trends (Supplementary Figure S4).

**Table 2:** ABT-888/MMS combination indices (CI) in WT vs PARP SNP rs1805407 carrier cell lines. CI values for three effect levels (ED_25_, ED_50_, ED_70_) representing low, medium, and high toxicity were calculated using median effect analysis by Chou and Talalay. For each cell line, combinations of ABT-888 and MMS were tested at fixed equipotency ratios calculated from single agent ED_50_ of MMS and ABT-888, as described in Materials and Methods. CI values <1, =1, and >1 are indicative of synergism, additivity and antagonism, respectively. Significant deviation from additivity was determined in Graph Pad Prism by a one sample t-test (two-tailed) using a hypothetical value of CI = 1.

Cell line	Genotype PARP1/SNP	Ratio ABT: MMS	Effect level	Combination Index^a^	Interpretation
A2780	C/T	1:3.8	ED25	0.08 ± 0.04, p < 0.001, 95% CI [−0.02, 0.18]	strong synergy
			ED50	0.22 ± 0.07, p = 0.003, 95% CI [0.04, 0.40]	strong synergy
			ED70	0.50 ± 0.09, p = 0.011, 95% CI [0.27, 0.73]	strong synergy
M14	C/T	1:2.3	ED25	0.65 ± 0.18, p = 0.081, 95% CI [0.20, 1.11]	mild synergy
			ED50	0.81 ± 0.10, p = 0.087, 95% CI [0.56, 1.07]	mild synergy
			ED70	0.98 ± 0.01, p = 0.093, 95% CI [0.95, 1.01]	additivity
H522	T/T	1:2.0	ED25	0.91 ± 0.33, p = 0.675, 95% CI [0.08, 1.73]	additivity
			ED50	1.21 ± 0.24, p = 0.269, 95% CI [0.61, 1.82]	antagonism
			ED70	1.30 ± 0.10, p = 0.011, 95% CI [1.30, 1.80]	antagonism
SW620	T/T	1:1.2	ED25	1.44 ± 0.04, p = 0.002, 95% CI [1.35, 1.52]	antagonism
			ED50	1.42 ± 0.08, p = 0.011, 95% CI [1.23, 1.61]	antagonism
			ED70	1.43 ± 0.09, p = 0.016, 95% CI [1.20, 1.66]	antagonism

## Discussion

Application of different therapies in well-defined subgroups of patients is an important step towards cancer precision medicine. A probabilistic graphical analytic framework identified ten gene expression and four DNA methylation variables that were directly linked to TMZ response that provided useful information about potential mechanisms. We also discovered a novel biomarker (PARP1 SNP rs1805407) of potentially high impact in stratifying patients in chemotherapy.

All patients carrying this mutation responded poorly to chemotherapy in our cohort. Since chemotherapy induces DNA damage and PARP1 is a key factor in DNA repair, the previous results led to the hypothesis that rs1805407 may be associated with improved DNA repair, which results in poor efficiency of the drug. Comparison of drug sensitivity values in WT and SNP carrier cell lines (NCI-60) showed four drugs with statistically significant differences in IC_50_ values, two of which were DNA damage agents. We found the WT cell lines to be more sensitive to these drugs. Follow-up *in vitro* experiments in cells from different cancer histologies showed that the sensitivity of SNP carriers to alkylating agent is increased after PARP inhibition. Furthermore, we showed that in SNP cell lines the combination treatment was synergistic, while in WT cell lines was at best additive or even antagonistic. We also note that this effect was independent of BRCA1 mutation since none of the tested cell lines carried this mutation. Since this SNP has high prevalence (24–32% and 65.5% in European and African populations, respectively) it has the potential of transforming the current chemotherapeutic strategies. SNP rs1805407 can be potentially used in the future as companion diagnostic marker for selecting the patients that are more likely to benefit from chemotherapy. Adequately powered future validation studies will test the clinical application of this SNP as response biomarker.

The mechanism of action of this SNP is currently unknown and is beyond the scope of this paper. However, one can postulate a possible scenario, based on publicly available data and what is known in the literature about PARP1 action and inhibition. PARP1 acts as a “molecular sensor” to identify DNA single strand breaks. Upon binding, PARP-1 undergoes autoPARylation and promotes PARylation of chromatin proteins, which recruit DNA repair factors^33–35^. Extensive autoPARylation of PARP1 results in dissociation from DNA, which is required for DNA repair completion^36^. It has been proposed that PARP1 inhibition acts on the autoPARylation stage, thus preventing PARP1 dissociation from the DNA (“PARP1-trapping”)^37,38^. PARP-DNA complexes are themselves cytotoxic and may underlie the differential efficacy of clinically relevant PARPi’s, which differ markedly in their PARP trapping potency^24^. Based on our findings we postulated that SNP rs1805407 affects PARP1 activity during treatment with alkylating agents. We found that SNP cell lines were sensitive to combination therapy with ABT-888, while WT in general were not. We subsequently conducted experiments with the FDA approved PARPi olaparib in A2780 (SNP carrier) and SW6220 (WT) cells and the results were more profound than with ABT-888. These are very important results as SNP rs1805407 can be potentially used in the future to decide whether a patient will receive a combination therapy or not.

In summary, we showed that (1) SNP carrying metastatic melanoma patients have poor response to chemotherapy; and (2) SNP carrying cells have increased sensitivity to combination treatment (alkylating agent + PARPi). Both these results are consistent with the theory that the identified SNP rs1805407 is associated with higher PARP1 expression. In this case, cancer cells in SNP carrying patients will repair the DNA damage, caused by chemotherapy, more efficiently. At the same time, inhibition of PARP-1 in SNP carrying cells can lead to higher number of PARP-DNA complexes, which will increase cytotoxicity. Supportive of the hypothesis of increased PARP1 expression in SNP carrying cells is the fact that rs1805407 is in perfect linkage disequilibrium with two PARP1 promoter SNPs (rs2077197 and rs6665208), which overlap ChIP peaks of cancer transcription factors. Based on GTEx data, rs1805407 is indeed an eQTL for PARP1.

In addition, we note that SNP rs1805405, which is in perfect linkage disequilibrium with our rs1805407, causes a mis-splicing event leading to increased expression of the shorter PARP1 form, PARP1-003. PARP1-003 includes only exons 1 and 2, thus coding only for the first zinc finger (ZF1) of PARP1. In PARP1 protein, ZF1 is responsible for recognition of DNA lesions and for initiation of DNA repair^39–41^. However, its overexpression can lead to inhibition of DNA repair^42^. Also, the short isoform uniquely maintains the auto-modification domain, which is most critical to PARP trapping. Experiments on measuring PARP1 trapping cannot be conducted in the context of this study, because cell lines have drastically different baseline response to alkylating agents (presumably due to differences in mutations in the DNA repair mechanism, permeability, etc) and -in addition- PARP1 is induced by alkylating agents (presumably at different levels in different cell lines). A future detailed analysis in cell lines or mouse models will shed more light in the mechanisms in which SNP rs1805407 may affect chemotherapy response.

We also found that patients with one or two alleles of rs1805407 in TCGA have significantly increased relative abundance of isoform PARP1-003 vs PARP1-001 (the full length mRNA), but the difference was small in absolute numbers. However, TCGA data are generated from pre-treatment samples, generally from primary tumors, and in melanoma predominantly from metastatic sites (also, pre-treatment). PARP1 expression is increased by DNA damage (treatment). The presence of SNP rs1805405 may affect the splicing rates and relative abundances of PARP1 isoforms, thus altering their relative protein levels. This is relevant in patients, especially knowing that TMZ is utilized in regimens that span either 5 days or 21 days out of 28-day cycles. Since the focus of this paper is in the discovery of an important biomarker for chemotherapy, the exact mechanism of action was not fully elucidated. The above stated hypotheses can guide future experiments *in vitro* and in a clinical setting.

The accurate prediction of therapy outcomes based on the molecular characteristics of tumors may alter the current landscape of cancer therapy given that immunotherapy results in substantial objective responses in some patients only. One can therefore expect that the accurate patient stratification into therapy-response categories may allow the overall percentage of disease control to increase through the current therapeutic armamentarium. In addition, insights into the molecular mechanism that characterizes a therapy-resistant phenotype may usher in new strategies to overcome therapy resistance.

## Supplementary information

Supplementary Information
