# Supplementary material for: PARP1 rs1805407 Increases Sensitivity to PARP1 Inhibitors in Cancer Cells Suggesting an Improved Therapeutic Strategy
Source: Sci Rep. 2019 Mar 1;9:3309. doi: 10.1038/s41598-019-39542-2 (PMC6397203; doi:10.1038/s41598-019-39542-2)
Supplement: Supplementary file 1 — Supplementary Information [file 41598_2019_39542_MOESM1_ESM.pdf]

SUPPLEMENTARY METHODS, FIGURES, and TABLES

PARP1 rs1805407 Increases Sensitivity to PARP1 Inhibitors in Cancer Cells Suggesting an Improved Therapeutic Strategy

Authors: Irina Abecassis[1†], Andrew J Sedgewick[2,3†], Marjorie Romkes[1], Shama Buch[1], Tomoko Nukui[1], Maria G. Kapetanaki[4], Andreas Vogt[2,5], John M. Kirkwood[1], Panayiotis V. Benos[2,3,*], Hussein Tawbi[6,7*]

Running Title: PARP1 Variant Predicts PARP inhibitor Sensitivity

Affiliations:

[1]Division of Hematology/Oncology, University of Pittsburgh School of Medicine, Pittsburgh, Pennsylvania, USA.

[2]Department of Computational and Systems Biology, University of Pittsburgh School of Medicine, Pittsburgh, Pennsylvania, USA.

[3]Joint Carnegie Mellon University-University of Pittsburgh PhD Program in Computational Biology, Pittsburgh, Pennsylvania, USA.

[4]Division of Pulmonary, Allergy and Critical Care Medicine, Department of Medicine, University of Pittsburgh, Pittsburgh, Pennsylvania, USA.

[5] Drug Discovery Institute, University of Pittsburgh School of Medicine, Pittsburgh, Pennsylvania, USA.

[6]Department of Melanoma Medical Oncology, Division of Cancer Medicine, University of Texas MD Anderson Cancer Center, Houston, Texas, USA.

[7]Department of Investigational Cancer Therapeutics, Division of Cancer Medicine, University of Texas MD Anderson Cancer Center, Houston, Texas, USA.

*To whom correspondence should be addressed: benos@pitt.edu, HTawbi@mdanderson.org

†These authors contributed equally.

Brief description of the analysis procedure

We briefly present here the steps we followed for the data analysis of the combined omics datasets and the TMZ response variable. The analysis consisted of four steps.

1. Filtering step. If the dataset contains omics data with thousands to millions of variables a filtering step is needed to select those variables that are more likely to be relevant to the clinical problem. Possible methods for this filtering step include a variance filter, using prior knowledge to select genes or pathways that are likely to be involved in the disease of interest, or using a statistical filter to choose genes that are highly correlated with or predictive of certain disease features. Here we use a generalized correlation measure (described below) that is able to calculate correlation between pairs of variables that can be categorical or continuous. We selected the 1000 features most correlated with response to treatment using this measure.

2. Normalization step. Most methods that identify conditional dependencies between continuous variables require these variables to be normally distributed. This is not the case with some of the biomedical data. For example, RNA-seq data are distributed according to the negative binomial distribution (*1*). We normalize our continuous variables using the non-paranormal transformation (*2*). This method maps each data feature to a normal distribution which helps to satisfy the assumptions of normality in our subsequent methods and to allow us to learn a network over differently distributed data sources (*3*).

3. Learning undirected graph over mixed type variables. For this step, we used our recently published algorithm, MGM (*4*). In brief, the likelihood of a mixed graphical model (MGM) can be described as follows (*5*):

$$p(x, y, \Theta) \propto \exp\left(\sum_{s=1}^{p}\sum_{t=1}^{p} -\frac{1}{2}\beta_{st}x_s x_t + \sum_{s=1}^{p}\alpha_s x_s + \sum_{s=1}^{p}\sum_{j=1}^{q}\rho_{sj}(y_j)x_s + \sum_{j=1}^{q}\sum_{r=1}^{q}\varphi_{rj}(y_r, y_j)\right)$$

where β is the edge potential between each pair of continuous features, α is the node potential of a continuous feature, ρ is the edge potential between continuous and discrete features and φ is the edge potential between pairs of discrete features. Since computing the exact likelihood for this mixed model is computationally intractable, the use of the pseudolikelihood (*6*) is necessary. For this step we used the Lee and Hastie pseudolikelihood calculation (*5*):

$$\tilde{l}(\Theta|x, y) = -\sum_{s=1}^{p}\log p(x_s|x_{\setminus s}, y; \Theta) - \sum_{r=1}^{q}\log p(y_r|x, y_{\setminus r}; \Theta)$$

where

$$-\sum_{s=1}^{p}\log p(x_s|x_{\setminus s}, y; \Theta) = -\frac{1}{2}\left(log\beta_{ss} + \beta_{ss}\left(\sum_{j}\frac{\rho_{sj}(y_j)}{\beta_{ss}} - \sum_{t\neq s}\frac{\beta_{st}}{\beta_{ss}}x_t - x_s\right)^2\right)$$

and

$$-\sum_{r=1}^{q}\log p(y_r|x, y_{\setminus r}; \Theta) = -log\frac{exp\left(\sum_s \rho_{sr}(y_r)x_s + \varphi_{rr}(y_r, y_r) + \sum_{j\neq r}\varphi_{rj}(y_r, y_j)\right)}{\sum_{l=1}^{L_r} exp\left(\sum_s \rho_{sr}(l)x_s + \varphi_{rr}(l, l) + \sum_{j\neq r}\varphi_{rj}(l, y_j)\right)}$$

This pseudolikelihood is convex and efficiently computable. Learning is performed using accelerated proximal gradient methods implemented in TFOCS (*7*). We used Nesterov's 1983 method for optimization with a maximum of 700 iterations for our stability runs and 1000 iterations for all other runs. We used a modification to the Stability Approach to Regularization Selection (StARS) method (*8*) on the range $.1 < \lambda < .3$ subject to an instability threshold of .05 to select the value $\lambda = .2$ which we used to learn the model presented in the results.

4. Directionality assesment step. Undirected graphs learned over datasets produced by an underlying directed model tend to generate false positive edges. Indeed, when there is a "collider" in the true graph, X→Z←Y (i.e., X and Y are causing Z) then the learned undirected model will be X – Z – Y – X. This is because X and Y are dependent given Z or Dep(X, Y | Z). The false positive edge X – Y can be removed if we perform a conditional independence test over all possible subsets. For example, in the simple case of X→Z←Y, we will find that Ind(X, Y | Ø), and so the X – Y edge will be removed and correct orientation of the X→Z and Z←Y edges will be thus established. In addition for some additional undirected edge, Z - W, in the absence of the edges X – W and Y – W, we can infer the direction Z→W. This is because a Z←W true edge would have produced false positive edges X – W and Y – W. We call this the *directionality assessment* step. Algorithmically we follow the procedure for PC-Stable (*9*) except we start from the MGM graph rather than a fully connected graph, and since we do not assume acyclicity we only use orientation rule R1.

Generalized Correlation. In order to measure association between a continuous and categorical variable or two categorical variables we use the following strategy. We would like to calculate the equivalent of Pearson's product moment coefficient for each possible pairing of these variables. The general formula for Pearson's correlation between two vectors of observations, *X* and *Y,* with means μ_X and μ_Y and standard deviations σ_X and σ_Y is $r_{XY} = \frac{cov(X,Y)}{\sigma_X \sigma_Y}$ where covariance is defined as $cov(X,Y) = E[(X - \mu_X)(Y - \mu_Y)]$. This is a standard calculation for pairs of continuous variables because mean and standard deviation are well defined. For pairs of binary variables, these values are also well defined, and this formulation is called the Matthews' Correlation Coefficient. For categorical variables we can calculate the covariance on a category by category basis. So for a categorical *X* continuous *Y*, we can focus on *a*, one of the categories

of *X* when calculating a sample covariance: $cov(X_a, Y) = E\big[\big(X_a - \mu_{X_a}\big)(Y - \mu_Y)\big] = \frac{1}{N-1}\sum_{i=1}^{N}[(\mathbb{I}(X_i = a) - \hat{p}_a)(Y_i - \hat{\mu}_Y)]$ where $\mathbb{I}(X_i = a)$ is an indicator function that is 1 when $X_i = a$ and zero otherwise, and $\hat{p}_a = \frac{1}{N}\sum_{i=1}^{N}\mathbb{I}(X_i = a)$ or the empirical probability of observing a in X. Since $\mathbb{I}(X_i = a)$ is equivalent to a Bernoulli random variable now it is easy to see that the sample standard deviation is $\hat{\sigma}_{X_a} = \sqrt{\frac{N}{N-1}\hat{p}_a(1 - \hat{p}_a)}$. Similarly, if both X and Y are categorical we now look at each possible pairing of categories separately so $cov(X_a, Y_b) = \frac{1}{N-1}\sum_{i=1}^{N}[(\mathbb{I}(X_i = a) - \hat{p}_a)(\mathbb{I}(Y_i = b) - \hat{q}_b)]$ where $\hat{q}_b$ is the empirical probability of observing *b* in *Y*. So, in a discrete-continuous pair, we now have a vector for the covariance and a vector for the standard deviations corresponding to the different levels of the categorical variable, we use the l_2 norm to calculate a single score from these vectors (where X is categorical): $r_{XY} = \frac{\|cov(X,Y)\|_2}{\|\sigma_X\|\sigma_Y}$. In the discrete-discrete case we have two matrices corresponding to the possible pairs of levels in the two variables, and we combine them with the Frobenius norm: $r_{XY} = \frac{\|cov(X,Y)\|_F}{\|\sigma_X\sigma_Y\|_F}$. Both of these cases result in non-negative values so to make the continuous-continuous values comparable with the others we take the absolute value so scores for all pairs of edges fall on the interval [0,1].

One motivation for this approach is that these sample covariances turn out to be proportional to the partial gradients of negative log pseudolikelihood in a factorized (i.e. zero edges) MGM as described above with respect to the edge parameters and variable levels (see (*5*) supplement). Namely: $\frac{\partial\tilde{l}}{\partial\beta_{ij}} = -2 * (N-1) * cov(X,Y)$, $\frac{\partial\tilde{l}}{\partial\rho_{ij}(a)} = -2 * (N-1) * cov(X_a, Y)$ and $\frac{\partial\tilde{l}}{\partial\phi_{ij}(a,b)} = -2 * (N-1) * cov(X_a, Y_b)$ where *X* is the indexed by *i* and *Y* is indexed by *j* in

the MGM and the pairs of X and Y are continuous-continuous, discrete-continuous, and discrete-discrete respectively.

Computational analysis methods – Software availability

The MGM-Learn platform was developed in MATLAB and is available upon request. Undirected graphs are learned using the MATLAB code from http://www.stanford.edu/jdl17/learningmgm.html. For the non-paranormal normalization we used HUGE (*10*). To quantify PARP1 isoform abundances from paired-end reads of TCGA metastatic melanoma samples we used *kallisto* (*11*) using transcript definitions from Ensembl (*12*).

SNP imputation on TCGA samples and NCI-60 cell lines

NCI-60 data were obtained from Cell Miner in June 2013 (http://discover.nci.nih.gov/cellminer/). For those cell lines or TCGA samples for which the identity of SNP rs1805407 was not available we used imputation to infer its identity. Using SNAP (*13*) we found 51 SNPs to be in perfect linkage disequilibrium (LD) with rs1805407 ($R^2 = 1$). Of these, 9 variants were covered by the Affymetrix SNP Array 6.0 used by the TCGA. To determine the rs1805407 genotype in TCGA samples we used birdseed calls (*14*) from Affymetrix Genome-Wide Human SNP Array 6.0. Only samples with a birdseed confidence less than 0.1 or where all 9 SNPs in perfect LD agreed with the birdseed call were used.

LEGENDS FOR SUPPLEMENTARY FIGURES AND TABLES

Fig. S1. Association of SNP rs1805407 to response to TMZ treatment.

Fig. S2. eQTL association of SNP rs1805407 to PARP1 expression in whole blood. Data from GTEx; p-value 5.5e-06)

Fig. S3. Cytotoxic effect of ABT-888, MMS or in combination in SNP *vs* WT cell lines. *Left panel.* Dose-effect curves for MMS, ABT-888 and ABT-888 + MMS combination. A2780 **(A)**, M14 **(B)**, SW620 **(C)** and H522 **(D)** cells exposed to ABT-888, MMS, or the drug combination (ABT + MMS were combined at the molar ratio of their IC_{50} values in each specific cell line). *Right panel.* Fraction affected (Fa)-C.I. plots. Combination index (C.I.) values are plotted as a function of the fractional inhibition (Fa). For each cell line, the mean of three independent experiments is displayed. The Fa-C.I. plots indicate that the cytotoxic effects of the chemotherapeutic agent MMS is synergistically enhanced by the combination with PARPi (ABT-888) in the SNP cell lines, A2780 **(A)** and M14 **(B)** (C.I. <1). In contrast, in the WT cell lines, SW620 **(C)** and H522 **(D)**, the interaction between ABT-888 and MMS is antagonistic (C.I. >1).

Fig S4. Assessment of drug interactions by Bliss independence model. Data from the median effect studies were independently analyzed by the Bliss independence method (*15*). Growth inhibition curves of individual agents and their combinations were first fitted to a four parameter logistic equation. Affected fractions (Fa) for concentrations of individual drugs that corresponded to their respective concentrations in the combination were then interpolated and used to compute an expected level of activity (Fa) according to $Fa = Fa_{drugA} + Fa_{drugB} - Fa_{drugA} * Fa_{drugB}$ (*15*). Expected effect levels (black bars) were then compared to actual toxicity caused by the MMS/ABT-888 combination (gray bars). Observed effect levels that are larger than

expected constitute synergy; effect levels smaller than expected, antagonism. The data are largely consistent with the median effect analysis, showing synergy to additivity in the SNP cell lines, and additivity to antagonism in the WT cell lines.

Table S1. PARP1 SNPs in LD with rs1805407.

Table S2. Drug compounds with differential IC_{50} values on WT vs SNP cell lines for rs1805407. IC_{50} values derived from NCI60. Statistical significance was assessed with Wilcoxon rank sum test.

Table S3. PARP1 SNP rs1805407 genotyping analysis of a panel of human cancer cell lines. All six of the cell lines reported in the literature to be "resistant" to chemotherapy + PARPi combination treatment were WT for the rs1805407 locus. Six out of the nine cell lines reported to be "sensitive" had at least one copy of C in this locus. Cell line was considered "sensitive" when chemopotentiation ratio was ≥ 2. *S:* sensitive; *R:* resistant.

Table S4. Results from MMS treatment of cell lines with and without PARP1 inhibitor (ABT-888). The data from the MTT assays were expressed as mean ± standard deviation (SD). The ratio between the IC_{50} means of MMS treatment alone and in combination with ABT-888 was calculated for each cell line. A Potentiation factor (ratio) ≤ 1 indicates no chemo-potentiation.

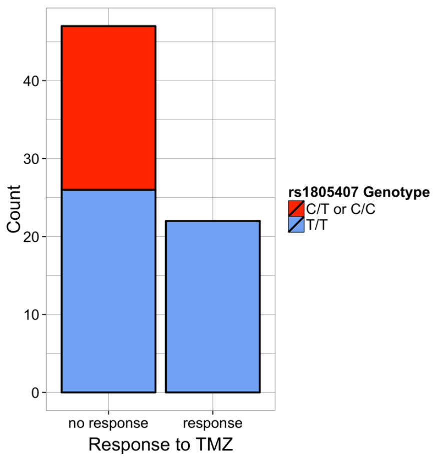

Supplementary Figure S1. Association of SNP rs1805407 to response to TMZ treatment.

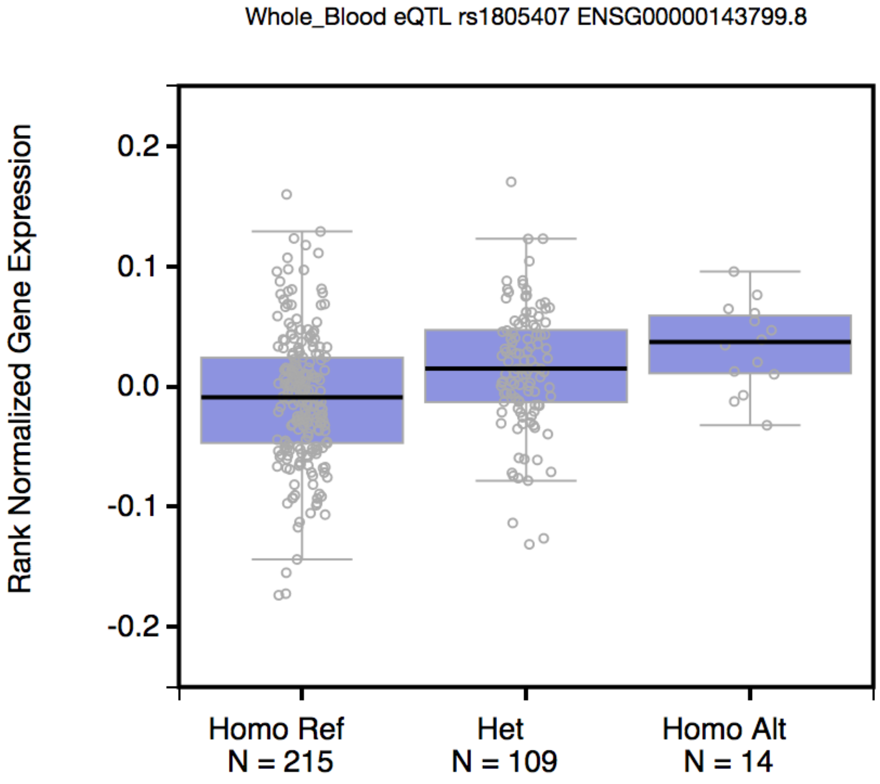

Supplementary Figure S2. eQTL association of SNP rs1805407 to PARP1 expression in whole blood. Data from GTEx; p-value 5.5e-06)

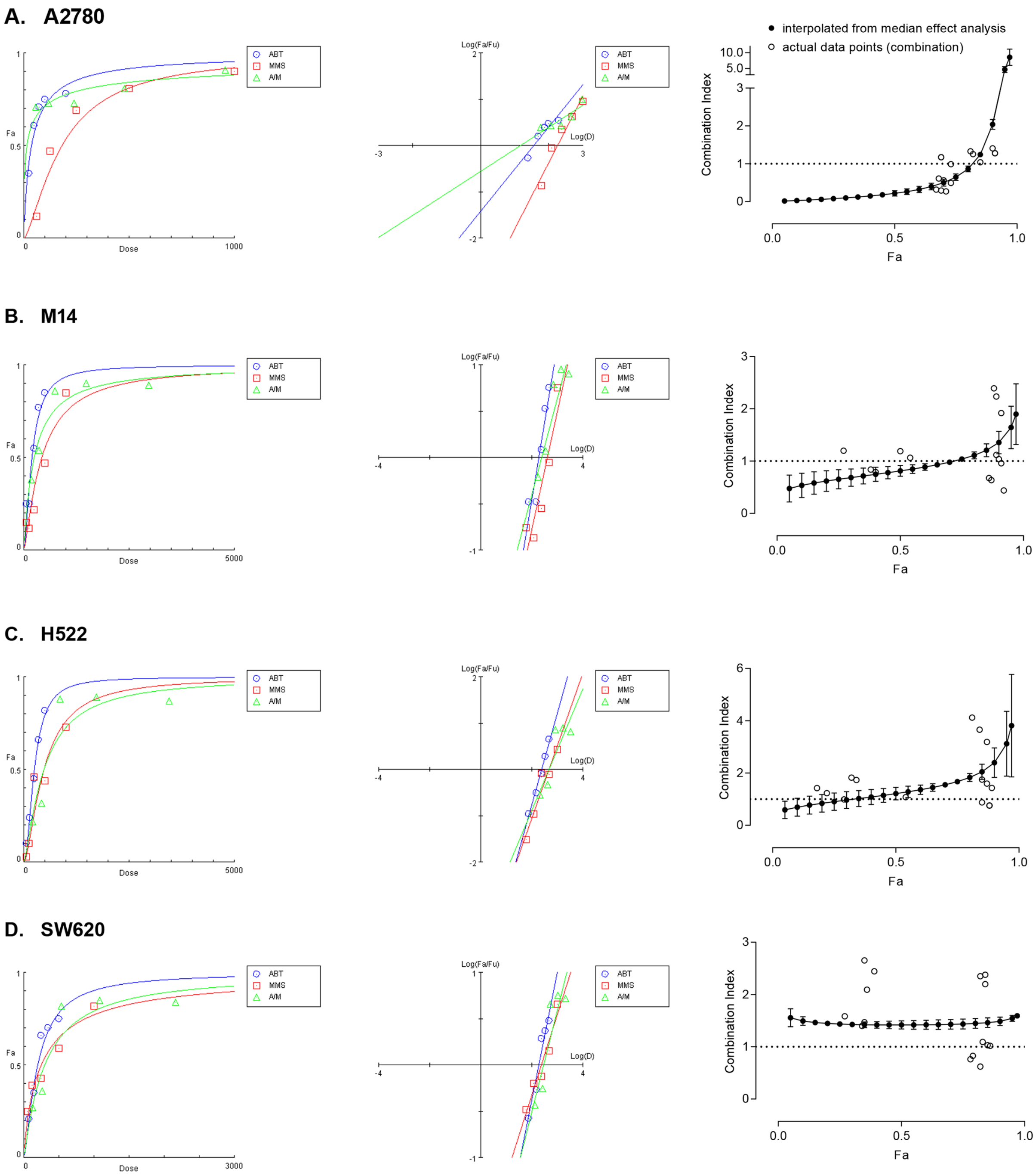

Supplementary Figure S3. Cytotoxic effect of ABT-888, MMS or in combination in SNP *vs* WT cell lines. *Left panel.* Dose-effect curves for MMS, ABT-888 and ABT-888 + MMS combinations. A2780 **(A)**, M14 **(B)**, SW620 **(C)** and H522 **(D)** cells were exposed to ABT-888, MMS, or combinations thereof at the molar ratio of their IC_{50} values in each specific cell line. *Middle panel.* Determination of median effect (Dm) and slope (m) from linearized inhibition curves. *Right panel.* Fraction affected (Fa)-CI plots. Combination indices were calculated for each effect level (closed symbols, *interpolated from median effect analysis)* and for each dose of the MMS/ABT-888 combination (open symbols, *actual data points (combination)*). Plots show

CI values as a function of the fraction affected (Fa). CI values of <1, 1 (solid line), and >1 indicate synergism, additivity, and antagonism, respectively. For the interpolated CI values, each data point represents the mean CI ± S.D. of three independent experiments; open circles are the individual data points for the combinations from the three independent repeats. The Fa-CI plots indicate that the cytotoxic effect of the chemotherapeutic agent MMS is synergistically enhanced (CI <1). by the combination with PARPi (ABT-888) in the SNP cell lines, A2780 **(A)** and M14 over a wide range of effect levels. **(B)** In contrast, in the WT cell lines, SW620 **(C)** and H522 **(D)**, the interaction between ABT-888 and MMS is mostly antagonistic (CI >1).

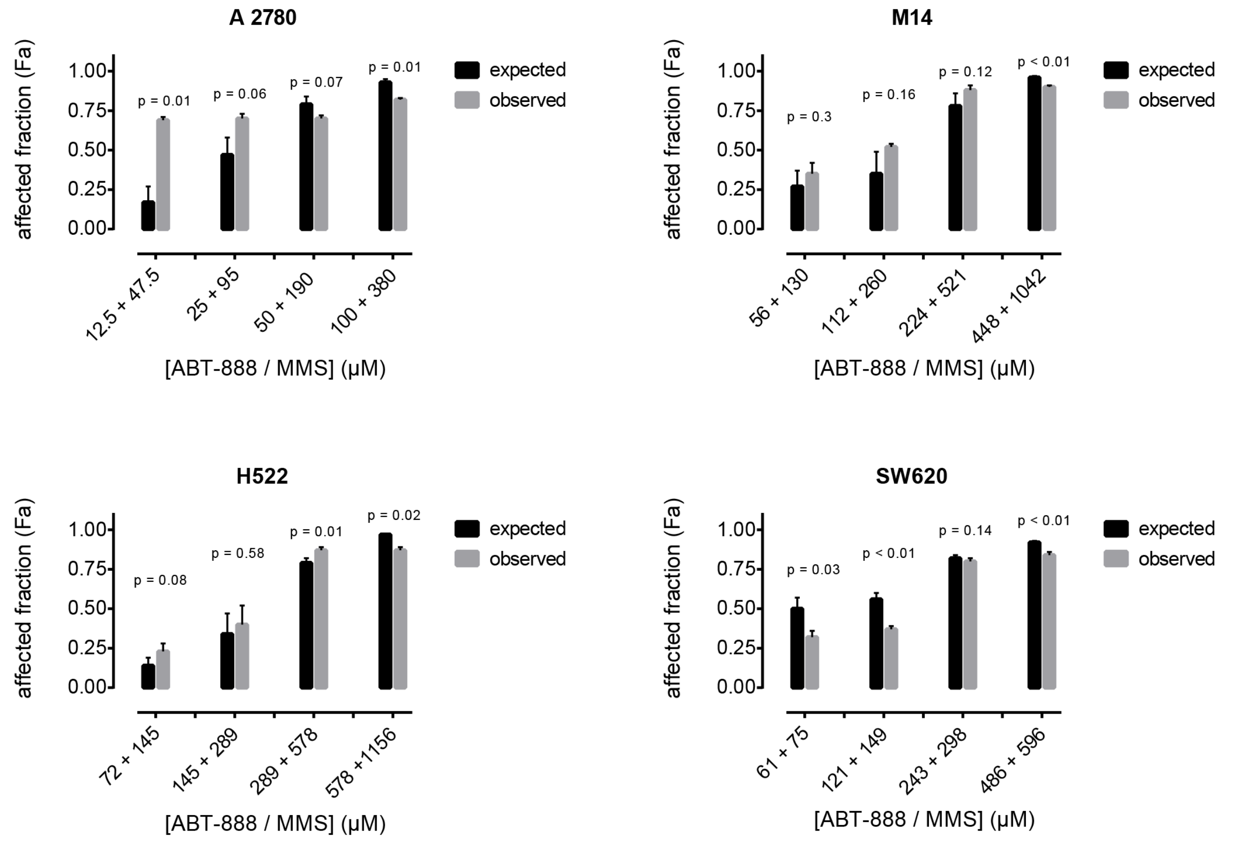

Supplementary Figure S4. Assessment of drug interactions by Bliss independence model. Data from the median effect studies were independently analyzed by the Bliss independence method (*15*). Growth inhibition curves of individual agents and their combinations were first fitted to a four parameter logistic equation. Affected fractions (Fa) for concentrations of individual drugs that corresponded to their respective concentrations in the combination were then interpolated and used to compute an expected level of activity (Fa) according to $Fa = Fa_{drugA} + Fa_{drugB} - Fa_{drugA} * Fa_{drugB}$ (*15*). Expected effect levels (black bars) were then compared to actual toxicity caused by the MMS/ABT-888 combination (gray bars). Observed effect levels that are larger than expected constitute synergy; effect levels smaller than expected, antagonism. The data are largely consistent with the median effect analysis, showing synergy to additivity in the SNP cell lines, and additivity to antagonism in the WT cell lines.

Supplementary Table S1. PARP1 SNPs in LD with rs1805407.

SNP	Distance	R^2	D'	Chr	Coord_hg18	GeneVariant
rs3219031	437	1	1	chr1	224656019	INTRONIC
rs3219027	580	1	1	chr1	224657036	INTRONIC
rs6701634	1792	1	1	chr1	224654664	INTRONIC
rs3754370	2445	1	1	chr1	224658901	INTRONIC
rs3768347	2912	1	1	chr1	224659368	INTRONIC
rs3768346	3021	1	1	chr1	224659477	INTRONIC
rs7522351	3435	1	1	chr1	224659891	INTRONIC
rs7525191	3438	1	1	chr1	224659894	INTRONIC
rs4653732	4273	1	1	chr1	224660729	INTRONIC
rs10799349	4317	1	1	chr1	224652139	INTRONIC
rs7542788	4530	1	1	chr1	224651926	INTRONIC
rs7548007	4553	1	1	chr1	224651903	INTRONIC
rs4653733	4780	1	1	chr1	224661236	INTRONIC
rs60698376	5024	1	1	chr1	224661480	N/A
rs4653731	5861	1	1	chr1	224650595	INTRONIC
rs2077197	6206	1	1	chr1	224662662	UPSTREAM
rs12240196	6350	1	1	chr1	224650106	INTRONIC
rs59672299	7760	1	1	chr1	224664216	N/A
rs1073991	8759	1	1	chr1	224647697	INTRONIC
rs2136876	8880	1	1	chr1	224647576	INTRONIC
rs1000033	9446	1	1	chr1	224647010	INTRONIC
rs6665208	9541	1	1	chr1	224665997	UPSTREAM
rs1002153	9646	1	1	chr1	224646810	INTRONIC
rs2280712	9740	1	1	chr1	224646716	INTRONIC
rs1805405	9812	1	1	chr1	224646644	SPLICE_SITE, INTRONIC
rs6679573	11114	1	1	chr1	224667570	INTERGENIC
rs10915987	11848	1	1	chr1	224668304	INTERGENIC
rs3219043	12239	1	1	chr1	224644217	INTRONIC
rs77173384	12382	1	1	chr1	224668838	N/A
rs28407557	12564	1	1	chr1	224669020	INTERGENIC
rs4653445	12927	1	1	chr1	224643529	INTRONIC
rs2293464	13537	1	1	chr1	224642919	INTRONIC
rs12068460	13912	1	1	chr1	224670368	INTERGENIC
rs3219053	15279	1	1	chr1	224641177	INTRONIC
rs1805408	16431	1	1	chr1	224640025	INTRONIC
rs3219058	17039	1	1	chr1	224639417	INTRONIC
rs6681537	19603	1	1	chr1	224676059	INTERGENIC
rs3219073	20458	1	1	chr1	224635998	INTRONIC
rs2271343	22270	1	1	chr1	224634186	INTRONIC

rs732284	22825	1	1	chr1	224633631	INTRONIC
rs3219115	32892	1	1	chr1	224623564	INTRONIC
rs752308	38327	1	1	chr1	224618129	INTRONIC
rs747658	38655	1	1	chr1	224617801	INTRONIC
rs747659	39092	1	1	chr1	224617364	INTRONIC
rs6664761	39642	1	1	chr1	224616814	INTRONIC
rs2282400	42834	1	1	chr1	224613622	DOWNSTREAM
rs6675427	45851	1	1	chr1	224610605	DOWNSTREAM
rs6675327	45924	1	1	chr1	224610532	DOWNSTREAM
rs6661762	46142	1	1	chr1	224610314	DOWNSTREAM
rs1991865	48782	1	1	chr1	224607674	INTERGENIC
rs12092726	50806	1	1	chr1	224605650	INTERGENIC
rs3219023	1223	0.947	1	chr1	224657679	INTRONIC
rs7531668	6186	0.945	1	chr1	224662642	UPSTREAM
rs12025487	15060	0.945	1	chr1	224671516	INTERGENIC
rs1109032	28430	0.945	1	chr1	224628026	INTRONIC
rs3754375	28768	0.945	1	chr1	224627688	INTRONIC
rs4653735	10521	0.891	1	chr1	224666977	UPSTREAM
rs878367	52311	0.891	1	chr1	224604145	INTERGENIC
rs7527192	6246	0.838	1	chr1	224662702	UPSTREAM

Supplementary Table S2. Drug compounds with differential IC_{50} values on WT vs SNP cell lines for rs1805407. GI50 values derived from NCI-60. Statistical significance was assessed with Wilcoxon rank sum test. Carmustine and Cyclophosphamide are classical DNA damaging alkylating agents. Parthenolide, a compound that induces apoptosis in acute myelogenous leukemia (AML) and progenitor cells (*16*). Increased sensitivity was observed for Irofluven, an alkylating agent that inhibits DNA replication (*17*). For comparison purposes, we also added the PARP1 inhibitor Olaparib (which is not statistically significant when used as single agent).

NSC	Name	FDA status	u	p
26271	Cyclophosphamide	FDA approved	175	0.01
157035	Parthenolide	FDA approved	148	0.02
683863	Irofulven (Hydroxymethylacylfulvene)	FDA approved	267.5	0.02
409962	Carmustine	FDA approved	294	0.03
747856	Olaparib	FDA approved	342.5	0.64

Supplementary Table S3. Potentiation of response to chemotherapy or radiation combined with PARP inhibition (from literature). PARP1 SNP rs1805407 genotyping analysis of a panel of human cancer cell lines. All six of the cell lines reported in the literature to be "resistant" to chemotherapy + PARPi combination treatment were WT for the rs1805407 locus. Six out of the nine cell lines reported to be "sensitive" had at least one copy of C in this locus. Cell line was considered "sensitive" when chemopotentiation ratio was ≥ 2. *S:* sensitive; *R:* resistant.

**Cell line**	**Tumor type**	**Response**	**Tumor type**	**rs1805407 Genotype**	**Therapy type**	**PARPi agent**	**REFs**
**LoVo**	Colon	R	Colon	T/T	TMZ	NU1025/ NU1085, AG14361	(*18, 19*)
**SW620**	Colorectal	R	Colorectal	T/T	Irinotecan	ABT-888	(*20*)
**H522**	Lung	R	Lung	T/T	TMZ	NU1025/ NU1085	(*18*)
**HT-29**	Colon	R	Colon	T/T	TMZ	NU1025/ NU1085	(*18*)
**SKOV-3**	Ovarian	R	Ovarian	T/T	TMZ	NU1025/ NU1085	(*18*)
**LS174T**	Colon	S	Colon	T/T	TMZ	NU1025/ NU1085	(*18*)
**HCT-116**	Colon	S	Colon	T/T	Irinotecan	ABT-888	(*20*)
**MDA-MB-231**	Breast	R	Breast	T/T	TMZ	NU1025/ NU1085	(*18*)
**MCF-7**	Breast	S	Breast	T/T	TMZ	NU1025/ NU1085	(*18*)
**Calu-6**	Lung	S	Lung	C/T	TMZ	CEP-6800	(*21*)
**M14**	Melanoma	S	Melanoma	C/T	TMZ	3-aminobenzamide	(*22*)
**A549**	Lung	S	Lung	C/T	TMZ	NU1025/ NU1085, AG1436	(*18, 19*)
**H460**	Lung	S	Lung	C/T	Radiation	MK-4827	(*23*)
**SK-Mel-2**	Melanoma	S	Melanoma	C/C			
**A2780**	Ovarian	S	Ovarian	C/T	TMZ	NU1025/ NU1085	(*18*)

Supplementary Table S4. Results from MMS treatment of cell lines with and without PARP1 inhibitor (ABT-888 or olaparib). The data from the MTT assays were expressed as mean ± standard deviation (SD). A "potentiation factor", defined as the ratio between the IC_{50} means of MMS treatment alone and in combination with ABT-888 or olaparib was calculated for each cell line. A potentiation factor (ratio) ≤ 1 indicates no chemo-potentiation.

ABT-888 (10 nM):

**Cell line**	**Tissue origin**	**PARP1/SNP genotype**	**MMS IC_{50}** (μM)	**MMS + ABT-888 IC_{50}** (μM)	**Potentiation factor**	***p*-value:**
**FEMX**	melanoma	**T/T**	166.3 (± 20.2)	176.0 (± 40.4)	0.945	**0.626**
**A375**	melanoma	**T/T**	306.0 (± 22.1)	283.3 (± 33.5)	1.080	**0.172**
**H-522**	lung	**T/T**	577.7 (± 56.8)	745.3 (± 68.6)	0.775	**0.147**
**SW620**	colon	**T/T**	299.4 (± 37.0)	449.4 (± 89.1)	0.666	**0.047**
**MDA-MB-231**	breast	**T/T**	287.2 (± 28.7)	303.2 (± 45.1)	0.947	**0.530**
**M14**	melanoma	**C/T**	520.8 (± 63.4)	359.8 (± 56.7)	1.447	**0.005**
**A549**	lung	**C/T**	254.8 (± 23.9)	143.9 (± 37.8)	1.771	**0.002**
**A2780**	ovarian	**C/T**	190.0 (± 41.0)	80.8 (± 14.7)	2.351	**0.003**
**H460**	lung	**C/T**	227.0 (± 21.4)	134.9 (± 20.5)	1.682	**0.002**

Olaparib (5 nM):

**Cell line**	**Tissue origin**	**PARP1/SNP genotype**	**MMS IC_{50}** (μM)	**MMS + olaparib IC_{50}** (μM)	**Potentiation factor**	***p*-value**
**SW620**	colon	**T/T**	342.7 (±68.7)	357 (±55.6)	0.960	**0.720**
**A2780**	ovarian	**C/T**	182.4 (± 24.0)	39.2 (± 8.8)	4.651	**0.017**

REFERENCES

1. S. Anders, W. Huber, Differential expression analysis for sequence count data. *Genome Biol* **11**, R106 (2010).
2. H. Liu, J. Lafferty, L. Wasserman, The nonparanormal: Semiparametric estimation of high dimensional undirected graphs. *The Journal of Machine Learning Research* **10**, 2295-2328 (2009).
3. K. Puniyani, E. P. Xing, NP-MuScL: unsupervised global prediction of interaction networks from multiple data sources. *Journal of computational biology : a journal of computational molecular cell biology* **20**, 892-904 (2013).
4. A. J. Sedgewick, I. Shi, R. M. Donovan, P. V. Benos, Learning mixed graphical models with separate sparsity parameters and stability-based model selection. *BMC Bioinformatics* **17 Suppl 5**, 175 (2016).
5. J. Lee, T. Hastie, Structure Learning of Mixed Graphical Models. *Journal of Machine Learning Research* **31**, 388-396 (2013).
6. J. Besag, Statistical analysis of non-lattice data. *J R Stat Soc D* **24**, 179-195 (1975).
7. S. R. Becker, E. J. Candès, M. C. Grant, Templates for convex cone problems with applications to sparse signal recovery. *Math. Prog. Comp.* **3**, 165-218 (2011).
8. H. Liu, K. Roeder, L. Wasserman, in *Advances in Neural Information Processing Systems*. (2010), pp. 1432-1440.
9. D. Colombo, M. H. Maathuis, Order-independent constraint-based causal structure learning. *The Journal of Machine Learning Research* **15**, 3741-3782 (2014).
10. T. Zhao, H. Liu, K. Roeder, J. Lafferty, L. Wasserman, The huge Package for High-dimensional Undirected Graph Estimation in R. *The Journal of Machine Learning Research* **98888**, 1059-1062 (2012).
11. N. Bray, H. Pimentel, P. Melsted, L. Pachter, Near-optimal RNA-Seq quantification. *arXiv preprint arXiv:1505.02710*, (2015).
12. P. Flicek *et al.*, Ensembl 2014. *Nucleic acids research*, gkt1196 (2013).
13. A. D. Johnson *et al.*, SNAP: a web-based tool for identification and annotation of proxy SNPs using HapMap. *Bioinformatics* **24**, 2938-2939 (2008).
14. J. M. Korn *et al.*, Integrated genotype calling and association analysis of SNPs, common copy number polymorphisms and rare CNVs. *Nature genetics* **40**, 1253-1260 (2008).
15. C. I. Bliss, The Toxicity of Poisons Applied Jointly. *Annals of Applied Biology* **26**, 585-615 (1939).
16. M. L. Guzman *et al.*, The sesquiterpene lactone parthenolide induces apoptosis of human acute myelogenous leukemia stem and progenitor cells. *Blood* **105**, 4163-4169 (2005).
17. Y. Wang, T. Wiltshire, J. Senft, E. Reed, W. Wang, Irofulven induces replication-dependent CHK2 activation related to p53 status. *Biochemical pharmacology* **73**, 469-480 (2007).
18. C. A. Delaney *et al.*, Potentiation of temozolomide and topotecan growth inhibition and cytotoxicity by novel poly(adenosine diphosphoribose) polymerase inhibitors in a panel of human tumor cell lines. *Clin Cancer Res* **6**, 2860-2867 (2000).
19. C. R. Calabrese *et al.*, Anticancer chemosensitization and radiosensitization by the novel poly(ADP-ribose) polymerase-1 inhibitor AG14361. *J Natl Cancer Inst* **96**, 56-67 (2004).
20. D. Davidson, Y. Wang, R. Aloyz, L. Panasci, The PARP inhibitor ABT-888 synergizes irinotecan treatment of colon cancer cell lines. *Invest New Drugs* **31**, 461-468 (2013).
21. S. J. Miknyoczki *et al.*, Chemopotentiation of temozolomide, irinotecan, and cisplatin activity by CEP-6800, a poly(ADP-ribose) polymerase inhibitor. *Molecular cancer therapeutics* **2**, 371-382 (2003).
22. L. Tentori *et al.*, Inhibition of telomerase increases resistance of melanoma cells to temozolomide, but not to temozolomide combined with poly (adp-ribose) polymerase inhibitor. *Molecular pharmacology* **63**, 192-202 (2003).
23. L. Wang *et al.*, MK-4827, a PARP-1/-2 inhibitor, strongly enhances response of human lung and breast cancer xenografts to radiation. *Invest New Drugs* **30**, 2113-2120 (2012).
